# Look how far we have come: BREAST cancer detection education on the international stage

**DOI:** 10.3389/fonc.2022.1023714

**Published:** 2023-01-04

**Authors:** Phuong Dung (Yun) Trieu, Claudia R. Mello-Thoms, Melissa L. Barron, Sarah J. Lewis

**Affiliations:** ^1^ Discipline of Medical Imaging Sciences, School of Health Sciences, Faculty of Medicine and Health, The University of Sydney, Sydney, NSW, Australia; ^2^ Department of Radiology, Carver College of Medicine, University of Iowa, Iowa City, IA, United States

**Keywords:** breast cancer, screening mammography, diagnostic accuracy, training & development, early detection

## Abstract

The development of screening mammography over 30 years has remarkedly reduced breast cancer–associated mortality by 20%-30% through detection of small cancer lesions at early stages. Yet breast screening programmes may function differently in each nation depending on the incidence rate, national legislation, local health infrastructure and training opportunities including feedback on performance. Mammography has been the frontline breast cancer screening tool for several decades; however, it is estimated that there are 15% to 35% of cancers missed on screening which are owing to perceptual and decision-making errors by radiologists and other readers. Furthermore, mammography screening is not available in all countries and the increased speed in the number of new breast cancer cases among less developed countries exceeds that of the developed world in recent decades. Studies conducted through the BreastScreen Reader Assessment Strategy (BREAST) training tools for breast screening readers have documented benchmarking and significant variation in diagnostic performances in screening mammogram test sets in different countries. The performance of the radiologists from less well-established breast screening countries such as China, Mongolia and Vietnam were significant lower in detecting early-stage cancers than radiologists from developed countries such as Australia, USA, Singapore, Italy. Differences in breast features and cancer presentations, discrepancies in the level of experiences in reading screening mammograms, the availability of high-quality national breast screening program and breast image interpretation training courses between developed and less developed countries are likely to have impact on the variation of readers’ performances. Hence dedicated education training programs with the ability to tailor to different reader cohorts and different population presentations are suggested to ameliorate challenges in exposure to a range of cancer cases and improve the interpretation skills of local radiologists. Findings from this review provide a good understanding of the radiologist’ performances and their improvement using the education interventions, primarily the BREAST program, which has been deployed in a large range of developing and developed countries in the last decade. Self-testing and immediate feedback loops have been shown to have important implications for benchmarking and improving the diagnostic accuracy in radiology worldwide for better breast cancer control.

## Introduction

Breast cancer is classified as the most common malignancy and the leading cause of cancer-related morbidity and mortality for women over the world. It has become a severe health problem as accounting for a third of all new cancer cases diagnosed among females (1). With advances in technology attributing to earlier diagnoses, as well as changes in environmental and lifestyle factors, an increasing trend of breast cancer incidence has been observed from developed countries in North America, Europe and Australia, as well as in developing countries across the Pacific region and towards Asia and Africa (2). Although there has been an increase in the number of new breast cancer cases detected worldwide annually, the prevalence of this disease is relatively low in both high income and middle/low-income countries. The average risk of a Caucasian woman in the United States or Australia developing breast cancer in her lifetime is approximately 13%. This means there is a 1 in 8 chance a women will develop breast cancer (3). With Mongoloid and Negroid women originally from Asia and Africa, this rate is estimated lower at 10%-11%. Therefore, only a small number of cancer cases are detected regardless of the large number of breast screening cases performed each year.

Examining mammograms in a screening environment requires expertise in image interpretation as detecting small and early signs of cancer lesions is more complex than diagnosing cancer in patients presenting with advanced stages. Developed countries that have an established nationwide/population-based breast screening program include Australia (4), the Netherlands (5), the United Kingdom (6) and the United States of America (7). These countries have regulations that require radiologists and other reader types to participate in continuing medical education (CME) and training to maintain a high level of performance. For example, Australian radiologists who interpret screening mammograms are obliged to complete a 5-year registrar program that includes breast imaging interpretation curricula. Once registered, screening radiologists must read a minimum of 2000 screening mammograms per year, obtain at least 4 CME hours annually and participate in an audit once every 3 years (4). BREAST (Breastscreen REader Assessment Strategy) have been developed to help radiologists at all stages of their expertise development, from those who have minimal experience and less time dedicated to screening mammograms through to those who wish to continuously update and test their knowledge (8–10). BREAST provides radiologists and screen readers with the opportunity to self-assess and improve their diagnostic performance in a simulated but highly authentic environment. This article aims to review international trends in current breast cancer status and a review of published educational tools that specifically related to breast cancer detection *via* mammograms that are available across a range of countries. Through this, an assessment of the effectiveness of the BREAST interactive training programs to improve radiologists’ diagnostic efficacy for early breast cancer detection is undertaken.

## Breast cancer: Incidence and mortality rates

**Figure 1:**
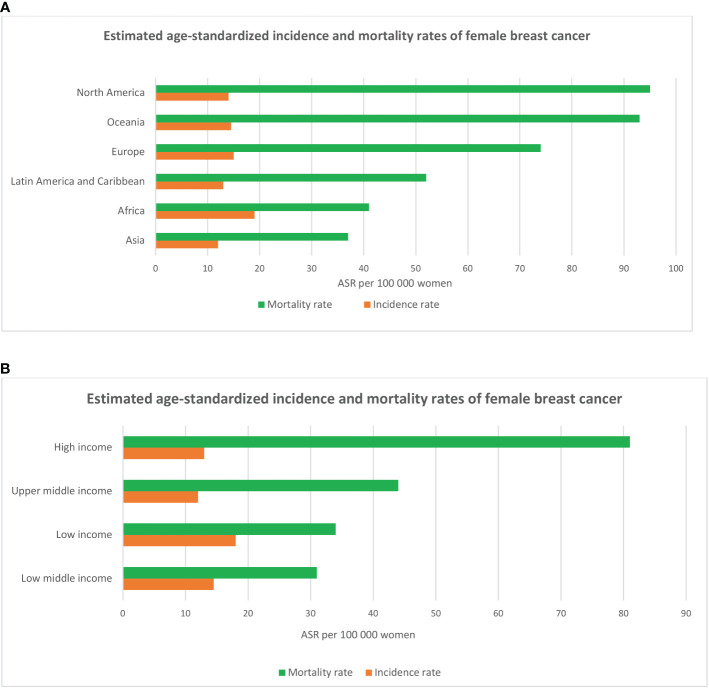
Estimated breast cancer age-standardized incidence and mortality rates across six continents **(A)** and in regions with different levels of income **(B)** according to the statistics of the International Agency for Research on Cancer (IARC - WHO) in 2020.

Breast cancer is the most common solid organ oncology presentation for women, with over 2.2 million new cancer cases worldwide in 2020, contributing to 24.5% of all cancer cases and almost 685,000 deaths, a 30% increase compared with the WHO statistics in 2012 (1, 11). Asian countries, representing 59.5% of the world’s population, make up the largest component, with 45.4% of new cases and 50.5% of deaths related to breast cancer. European countries, with 11% of the population, stand second with 23.5% of new cases and 20.7% of deaths. Although North America and Oceania represent only 8% of the global population, they account for 13.6% of new breast cancer cases and 8% of patients who die from this disease (1) (**Figure 1**).

Data from GLOBOCAN (WHO) in 2020 show that age-standardized breast cancer incidence rate was highest in high income countries (HIC) in Europe (Belgium, Netherlands, Luxembourg, France, Denmark, Finland, UK, Italy), Northern America (US, Canada), Oceania (Australia, New Zealand) and Asia (Singapore, Japan), ranging from 75 to 113 cases per 100,000 women. Mortality rates peak in low-middle-income (LMICs) and low-income countries (LICs) in Latin America and Caribbean (Barbados, Bahamas), Africa (Jamaica, Nigeria, Namibia, Ethiopia), and Asian Oceania (Fiji, Papua New Guinea, Malaysia, Thailand, Vietnam) with the range from 20 to 42 deaths per 100,000 women (age-standardized) (1). The high incidence of breast cancer in high-income countries has been described as reflecting the increase in the accessibility of mammography screening programs and the prevalence of well-known breast cancer risk factors (e.g sedentary lifestyles, late reproductive records and being overweight after menopause) (12–17), while high mortality rates in LMICs and LICs were found to be associated with lack of access to quality health care and treatment (18–22).

The incidence of breast cancer has steadily increased by an average of 1.4% per year for all age groups since 1990, based on the published report by the World Bank involving 185 countries across seven regions (23). This increase took place in more than 60% of nations experiencing socio-economic turmoil (24), whilst the data indicated that incidence rates had stabilized in HICs such as Canada, Europe, Australia and New Zealand (25) whereas in the US, stabilisation has been shown for white women but the incidence rate continues to increase for black and Hispanic women (26). The growth in the incidence rate among LICs and MICs are primarily due to an increase in risk factors associated with urbanization, including adopting western diets, obesity, lack of physical activity, early menarche (before age 12 years), late menopause (after 55 years old), delayed childbirth (after 30 years old) and a decrease in the number of children and shorter breastfeeding periods (27–30). For example, the obesity ratio in Australia and New Zealand in 2016 was approximately one to three adults, whilst obesity prevalence in Bangladesh, India and Vietnam was recorded as below 4%. However, there was a surge of 28% in the obesity rate in LICs and MLICs in Asia Pacific region from 2010 to 2016, with the increase particularly high at 50% among adults from 1.4% to 2.1% in Vietnam and 3.5% to 5.3% in Laos (31). Improved access to family planning initiatives in conjunction with socioeconomic growth between 1990s and 2000s has also led to a significant drop in fertility rates in Latin America, Africa and Asia from 5 – 7 births (1970s-1980s) to 1.5 - 3 births per woman (32).

Mortality rates from breast cancer have reduced over time ranging from 0.55% to 1.75% (from 20-26 per 100,000 women in 1990 to 17 per 100,000 women in 2017) in most HICs in Europe, Central Asia and North America, however it is consistently high and rising in many LMICs and LICs (23). The morality reduction in HICs is likely due to increasing early cancer detection by screening mammography programs and modern treatment methods, although the impacts of treatment on each individual may differ as well as the participation rate for routine screening alongside the accessibility of effective treatment programs. Contrary to the downtrend recorded in HICs, the uptrend in breast cancer mortality has been reported in Asia, Latin America, and Africa (25) plus within population sub-groups in some countries such as for black and Hispanic women in the US where the mortality rates are 28.4 per 100,000 women (26). A study comparing the data between 1990 and 2017 showed that the breast cancer mortality rate went up annually ranging from 0.36% per year in Middle East, North Africa to 0.56% in East Asia Pacific, Latin American Caribbean and Sub-Saharan Africa (23). It is described that the surge in breast cancer mortality in Japan that arose since the 1960s, is linked to the country undergoing a transition from a traditional Asian diet based on plant to a Western diet based on meat (this transition had occurred a decade earlier), which has been linked to the increase in obesity and overweight prevalence (33). Furthermore, even in some high-middle-income countries such as Malaysia and China, mammography screening has not yet been widely adopted at the population stage for various reasons such as sociocultural barriers, lack of equipment and clinician expertise and availability (34, 35).

The survival rate, which compares breast cancer mortality rates to incidence rates, was found to be lowest in less developed countries in Africa and South-Central Asia and highest in developed countries in North America, Europe and Oceania with the 5-year survival rate ranged from 53% in South Africa to 85% in Australia and 82% (Black women) and 92% white women in the US (26, 36). The low survival rate in low and middle-income countries highlights the fact that a large number of women were likely diagnosed in the late stages due to restricted or lack of screening programs and limited access to high-quality cancer treatment, in addition to insufficient staff and medical infrastructure including pathology services, radiotherapy units, and cancer treatment drugs (37). For example, in the period 2009 to 2010, over 75% of Nigerian breast cancer patients were detected with stage III or IV cancers (38), similarly to Vietnam, where 75% cancer cases were found to have local or distant metastasis (39). In contrast, high survival rates were observed in Northern America, Australia/New Zealand, Western and Northern Europe indicating low death rates in spite of high incidence rates as a consequence of early diagnosis and the availability of modern treatment methods (27). In HICs such as USA, Canada, UK, Australia and New Zealand, national breast screening programs are available, and women aged 50—75 are actively invited to have a free mammogram at set intervals, usually 1-3 years apart (40–42). Recalled women are frequently assessed with ultrasound, digital breast tomosynthesis or magnetic resonance imaging. However, optimal diagnostic and treatment methods for breast cancer are not commonly accessible in low-income populations. Efficient treatment is constrained by inadequate medical imaging equipment, including pathology and radiation therapy units and expensive cancer drugs (43). A systematic review highlighting radiotherapy capacity showed that there were more than 25 countries, mainly in Africa and Asia, that did not even have radiotherapy services (44). The International Atomic Energy Agency (IAEA) has anticipated a shortage of at least 5000 radiotherapy machines in developing nations (45). There are other barriers such as religious beliefs, cultural beliefs, and shame associated with breast cancer and undertaking treatment (46).

## Breast screening programs

An effective mammography screening program is a primary health service for detecting early abnormal lesions which will help to diminish the mortality risk from breast cancer for patients (40, 47). Digital mammography, with a considerably high specificity and sensitivity (over 90%) (48), is the main imaging tool used for breast cancer diagnosis and screening programs worldwide. Breast screening programs have been implementing for a number of decades in HICs with strong rates of successes. For instance, the UK has the National Health Service Breast Screening Programme which screened approximately 1.88 million women aged from 50 to 70 years old (73.4% participation rate from invitation) in 2010-2011, reported a cancer detection rate at 7.8 per 1000 women and 5-year survival for cancer patients of 85% (49). A similar result of the effectiveness of breast screening programs was found in Europe with a decrease of 25–30% breast cancer mortality for women between 50 and 74 years old. In Australia, the mortality rate has also decreased significantly since BreastScreen program began—from 74 deaths per 100,000 women in 1991 to less than 50 deaths per 100,000 since 2010 (40, 50). Overall, breast cancer screening recommendations are relatively similar across the HICs, with the most common age group targeted to be 50 to 70 years old for biannually screening. The American College of Radiology has the longest screening range, ranging from 45 to 75 years old with a suggestion for annual screening, whilst the UK has the longest screening interval time of 3 years (47).

The World Health Organization (WHO) estimates that up to 11 million cancer cases will be diagnosed in low- and middle-income countries by 2030, which is an 80% increase compared with 2008. By extrapolation, cancer will be the leading cause of death by the end of the 21^st^ century and is predicted to be the greatest obstacle for advancing human life expectancy (51). Early detection of cancer is one way to prevent death. However, screening for early signs of illness in asymptomatic patients is performed much less frequently in LICs and MICs than in HICs. Apart from lack of infrastructure as mentioned above, differences in breast characteristics among women in various populations can also influence the effectiveness of breast screening programs. Compared to Caucasian women (American, European, or Oceanian), Asian women have low breast cancer rates despite generally having small, dense breasts, and the mean onset age of breast cancer for Asian women is around 40–50 years old, which is 10 years younger than that for Caucasian women (46, 52). It is possible that some of the differences in risk profiles between Western and Asian women is related to the structure and gene expression profile of the normal breast. For example, normal breast epithelium is much more likely to be ER-positive in Caucasian women than in Japanese women (53). In addition, breast size is a highly heritable trait, with a twin study estimating the heritability of bra cup size to be 56% (54). Several genome-wide association studies have also identified common genetic variants associated with breast size (55, 56). Asian women typically have smaller breasts than women of Caucasian ancestry. A large cohort of 24,353 Singaporean women showed that the average bust line and total breast area was 91.2 cm and 102.3 cm^2^ (57) while the UK and Australian women were found with breast volumes calculated using the photograph-contours ranged from 90 to 1544 cm^3^ (58). Several demographics, reproductive and lifestyle factors have been suggested to influence breast size, but most of these links are anecdotal in nature. Variables found to be significantly associated with bust line and total breast area included Body Mass Index (BMI), marital status, and working status. Age, ethnicity, and number of children were significant predictors of breast area, but not bust line (57).

Additionally, Asian women have comparatively denser parenchyma when compared to Caucasian women, which in turn is related to a reduce efficacy with mammography screening for early cancer detection (46, 59). For example, Maskarinec et al. investigated variations in mammography densities between Japanese, Chinese and Caucasian (US) women and found that both Japanese and Chinese women had an average of 15% smaller unadjusted dense area, yet the proportion of breast density tissues was 20% higher than in Caucasian women (60). However, many of these studies were conducted in the early days of mammography when radiographers had limited experience in mammographic positioning and the equipment such as the compression paddles were not as developed or of high quality compared to present day equipment and techniques. In a recent study of 28231 Singaporean women undergoing screening mammography, the authors reported that the range of mammographic abnormalities was similar to the findings in the Caucasian population (61).

In many Asian countries, especially LICs, ultrasound has been considered as a good alternative for mammography in breast cancer screening, because of its advantage in women with high dense breasts, wide accessible and low operating costs (62). Nevertheless, there are also drawbacks related to ultrasound such as its accuracy dependent on the skills of the probe operator, it is less adept at detecting calcifications and can produce a higher rate of false positives than mammography (62, 63). Whether mammography screening programs should be implemented more widely in certain populations or LIC/MIC countries is a challenging concept. With resource-restricted healthcare systems, most LIC/MIC nations consider that “awareness of breast disease” may be a priority before conducting extensive population-based screening (64). However, significant economic growth and social development that has taken place in recent years, along with infrastructure and lifestyle changes, have led to many LICs and MICs to consider the introduction for formal mammography screening programs more widely.

**Figure 2:**
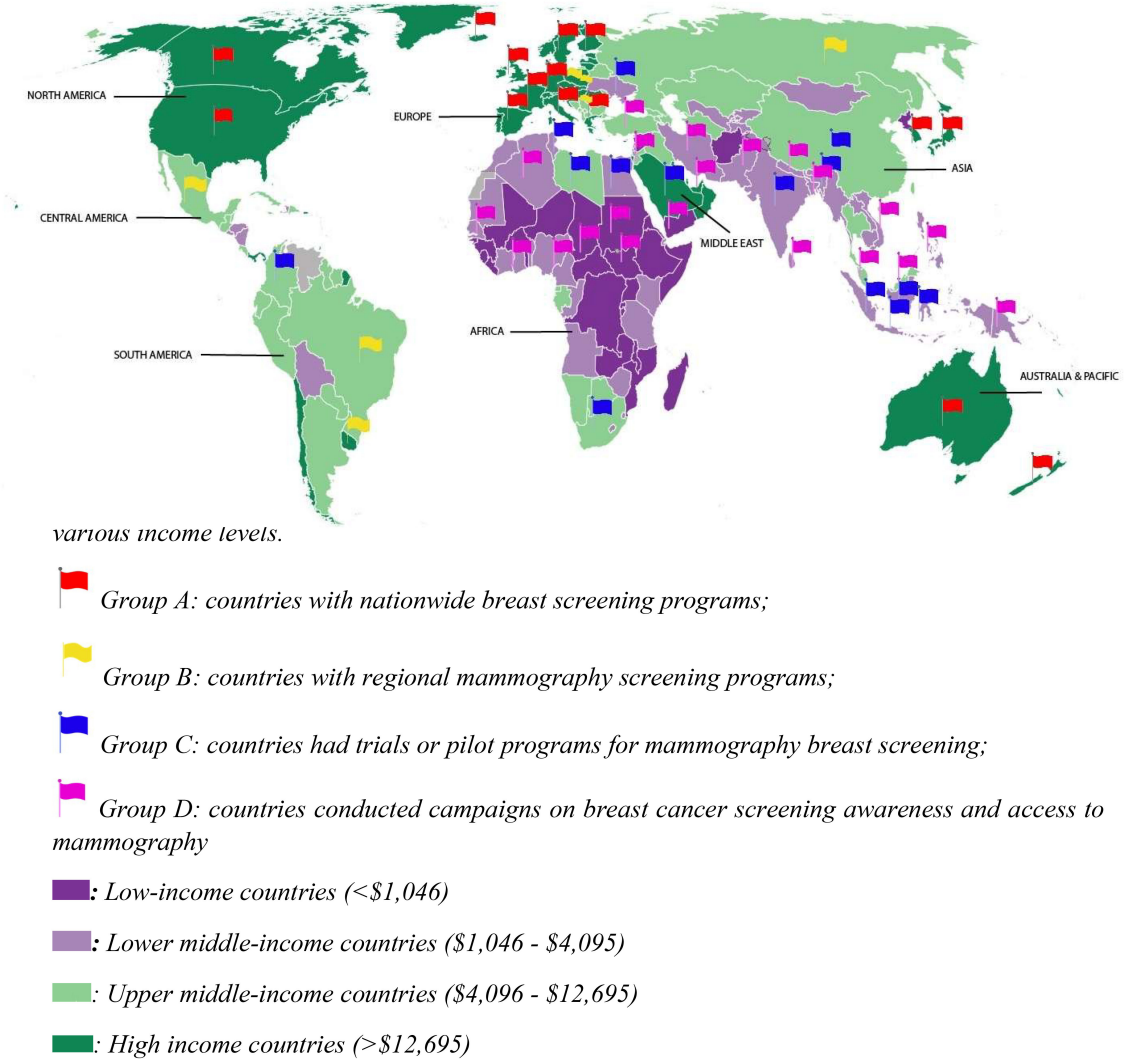
Distribution of mammography screening programs across continents and countries with various income levels.

There were six MIC countries (Russia, Brazil, Mexico, Uruguay, Hungary, Macedonia) which have nationwide or regional mammography screening programs with various recommendation for screening women from age 40 or 50 to 69 biennially. Screening participation rates in these countries, however, fluctuate considerably and is well below 70%, with modelling showing that a participation rate of 70% is optimal for breast cancer mortality reduction (65). Eight countries including South Africa, China, India, Indonesia, Colombia, Ukraine, Bulgaria, and Egypt conducted pilot studies to evaluate the diagnostic accuracy or cost effectiveness of a mammography screening program, which were aimed to notify policy makers practicability of a nationwide screening program. Yet the effectiveness of these pilots was not clear, so the implementation of national screening program is still on hold. One example was the trial from India which found that breast self-exam performed annually from age 40 to 60 had been almost as effective as biennial mammography screening in terms of reducing breast cancer mortality, while incurring only half of the total cost for a mammography screening program (66) suggesting that western mammography screening programs may not be cost-effective, especially given competing medical priorities and economic conditions. This may also be a feature of the population where higher breast density is documented for southern Asian/Indian women (**Figure 2**).

Other countries that have not published research on mammography screening occasionally provide population-based surveys for public awareness of breast cancer. In general, these surveys show that women in LICs are less aware of breast cancer and have been shown to have very low mammography utilization. For example, less than 20% of Iranian women have undertaken mammography (67). One survey in a less developed area of South Africa reported that no women at all have been screened using mammography (68). Although some countries have indicated their intention to introduce mammography screening programs, they are often referred to as “diagnostic mammography programs” after the mammogram has been identified as abnormal or after women who have experienced suspicious symptoms of breast cancer.

## Breast screening reader training programs and BREAST

For a mammography screening program to succeed, diagnostic accuracy plays a vital role. In HICs, diagnostic efficacy of breast screening readers (radiologists, breast physicians or reporting radiographers) who interpret the mammograms is regularly monitored through clinical audit programs (69), so that readers with low performance levels can be identified and obtain further training. Nevertheless, most screen readers are exposed to low number of cancer cases in a clinical practice because of breast cancer’s low incidence (approximately 8-15 cancers per 1000 screening women in HICs and even lower in MICs and LICs). Sensitivity and specificity are two of the most important parameters to assess the correct diagnosis of cancer and non-cancer in the population, however these metrics take time to collect due to screening intervals which range from 1-3 years in many established programs, and for *ad-hoc* screening, the interval period can be hugely variable. Realistically, clinical audit programs can take several years to collect sufficient data to classify reader performances against national standards. Once training programs are established, it may again take years for any progress in diagnostic performances to be identified.

**Figure 3:**
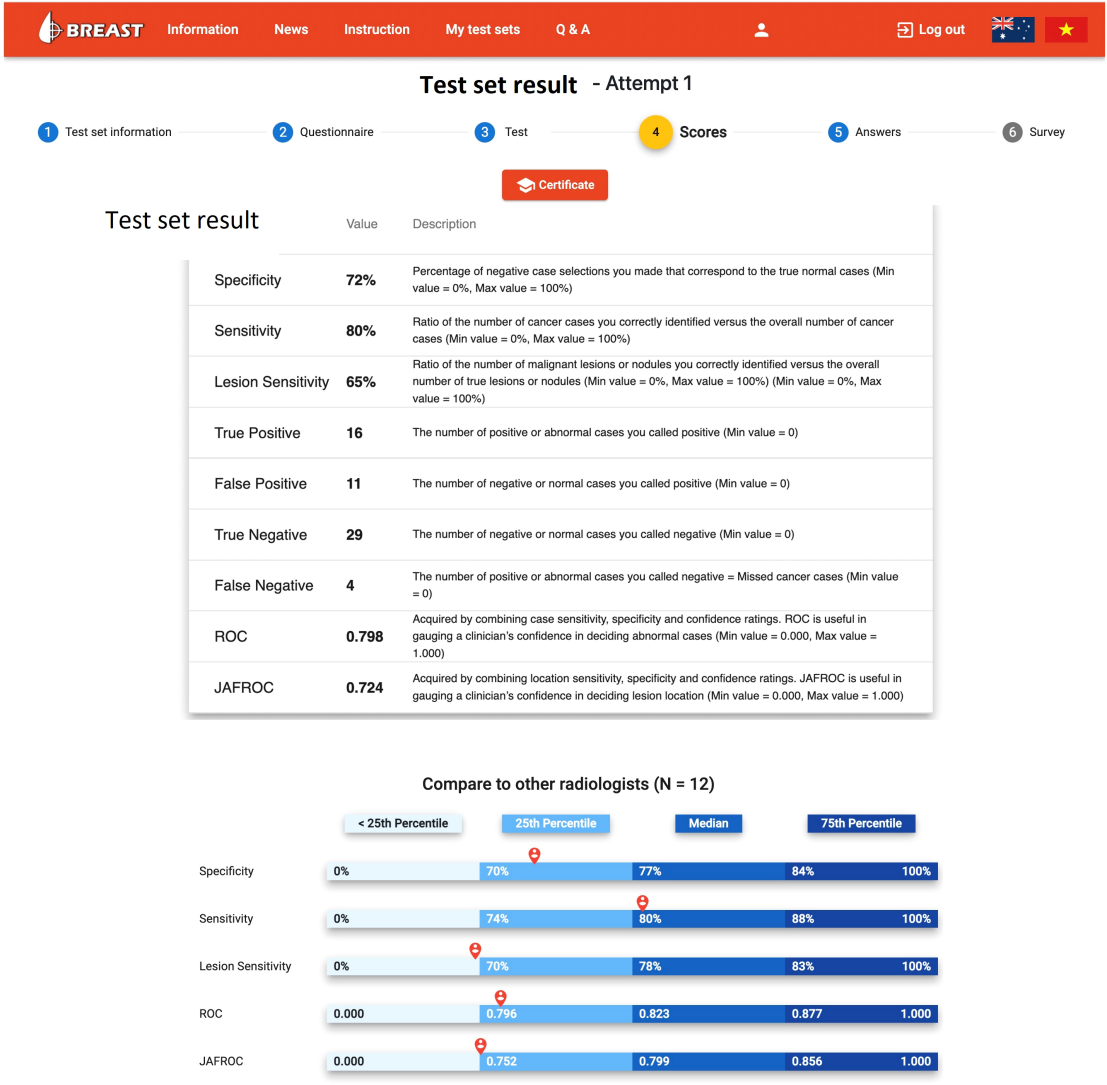
The diagnostic report on the BREAST platform to a radiologist when a mammogram test set is completed (www.breastaustralia.com).

**Figure 4:**
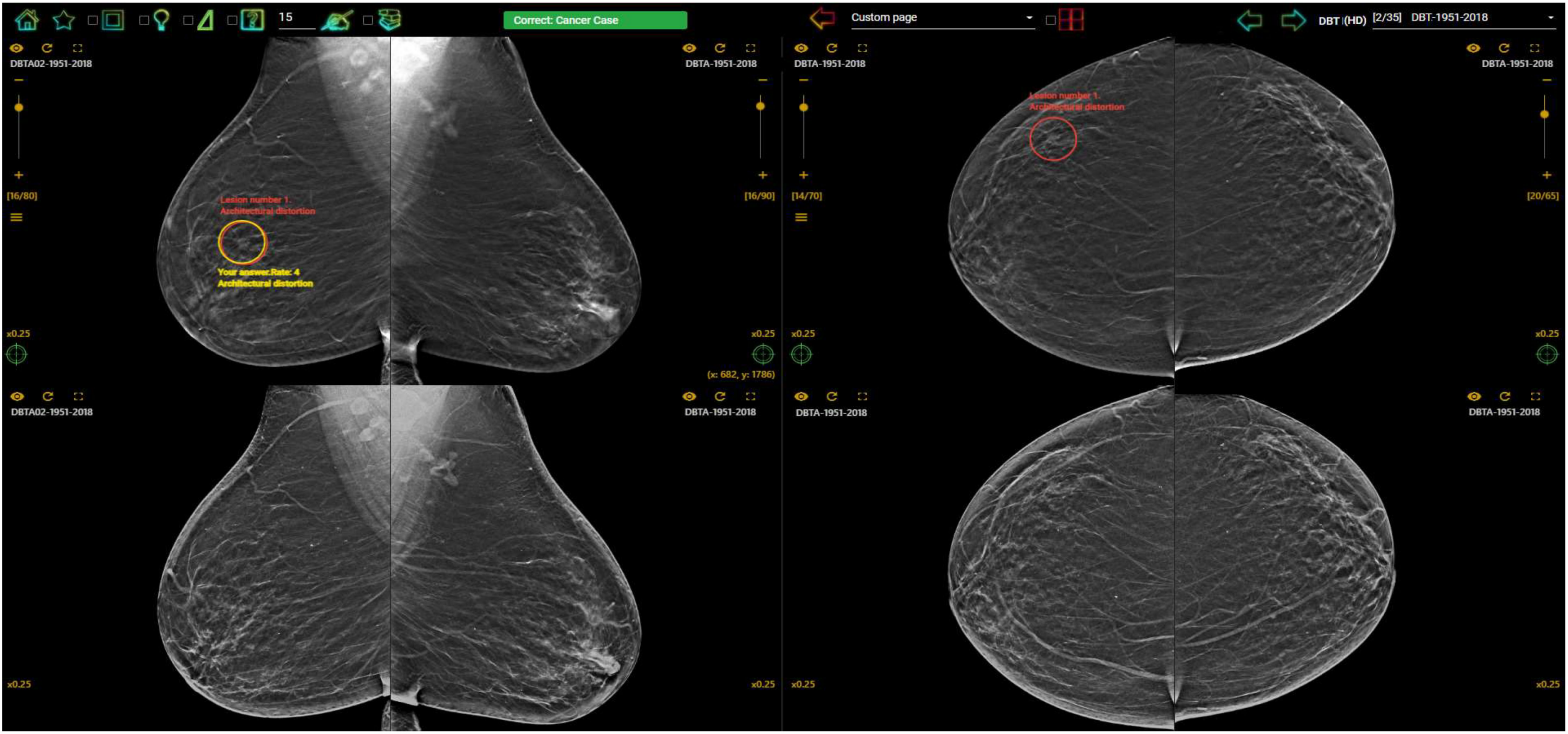
Feedback on the BREAST platform for a DBT case interpreted by a radiologist with a correct cancer location detection on the DBT slice (red circle (truth) and yellow circle (user’s marking) were overlapped) on RMLO (Right Mediolateral Oblique) view and a missed cancer location (red circle) on RCC (Right Craniocaudal) view. The first row displayed DBT images and synthesized views were shown on the second row (www.breastaustralia.com).

In countries without a breast screening program, the radiologists are even less likely to be exposure to early breast cancer cases on mammograms and thus be unaware about their diagnostic performance due to the lack of clinical audit data. Fortunately, there is a high demand for assessment and training programs with immediate feedback to identify and improve low performance readers, and this leads to the introduction and implementation of mammogram test set innovations such as the Breastscreen REader Assessment STrategy (BREAST) (8–10), PERFORMS (70) and Detected-X (71). These are novel web-based training solutions which present radiologists, breast physicians and radiology trainees (also known as registrars) with high-quality test sets of challenging mammographic examinations (Full Field Digital Mammography (FFDM) or Digital Breast Tomosynthesis (DBT) to interpret, and then provide scores and instant feedback on their diagnostic performances at the end of the test set where overall metrics such as sensitivity, specificity and ROC AUC (Area Under the Receiver Operating Characteristic Curve) can be calculated (**Figures 3**, **4**). Applying test sets to training platforms offers many benefits. Test sets could be arranged in such a way that the intervention is likely to explain for measured changes in diagnostic performances of radiologists. In addition, training sets are typically heavily enriched with pathology-proven cancer cases so have a much higher prevalence and results are almost immediately available to users.

**Figure 5:**
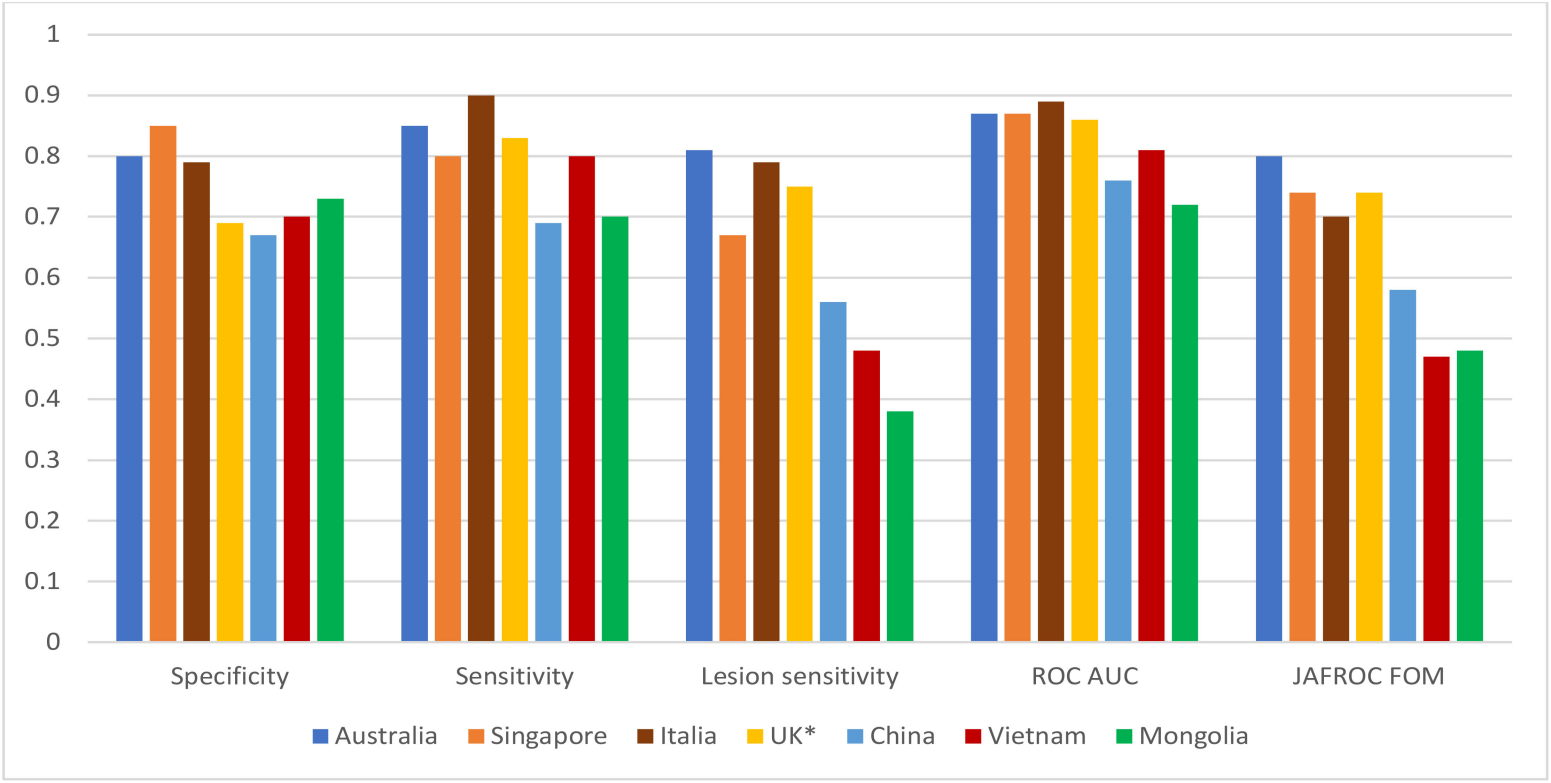
Diagnostic performances of radiologists in different countries in full-field digital mammogram BREAST test sets. *: Reporting radiographers.

Among a range of established education programs, BREAST has confirmed its usefulness and effectiveness through the largest number of publications in peer-reviewed journals with high impact factors. Between 2011 and 2021, BREAST has investigated diagnostic performances of radiologists, breast physicians and reporting radiographers in a variety of HIC, MIC and LIC countries with and without national breast screening programs including Australia, UK, Italy, Singapore, China, Mongolia, Iran and Vietnam *via* their mammogram-based test sets. The number of participants in the published studies have ranged from 10 to 117 and the number of mammographic cases included in test sets range from 35 to 60. Findings from studies show that radiologists from LIC countries with lack of national breast screening programs such as China, Mongolia, and Vietnam (72–74) displayed a significantly lower diagnostic accuracy in detecting cancer lesions on mammograms than radiologists from developed countries with well-established breast screening programs such as Australia, UK, Italy, Singapore (73, 75, 76) (**Figure 5**). The average differences in the performances between the two groups of countries (LIC versus HIC) were 8% in specificity (0.78 vs 0.70), 12% in sensitivity (0.85 vs 0.73), 29% in cancer location sensitivity (0.76 vs 0.47), 11% in ROC AUC (Area under the ROC Curve) (0.87 vs 0.76) and 24% in JAFROC FOM (Jackknife free-response receiver operating characteristic – figure of merit) (0.75 vs 0.51). This difference was not only reported in digital mammogram test sets but also found in DBT test sets. For example, in a recent study, it was found that the false positive and false negative rates of Chinese radiologists reading the DBT test set *via* the BEAST platform was 52% and 69% compared with 36% and 35% in Australian radiologists (77). This large difference in cancer detection accuracy might imply that a great number of cancer cases could be missed or incorrectly reported in the clinical practices among MIC and LIC countries, which could have harmful implications for treatment outcomes of patients.

In addition, BREAST studies reported findings based on performances of radiologists from different countries in reading mammograms with different level of breast density and the ability to detect various types of cancer appearances. The most challenging type of cancer lesions to detect on mammograms for LIC radiologists were small lesions such as stellate/spiculated masses along with architectural distortions (the missed rate was 55%-75%) (77, 78), while discrete masses and asymmetric density (or non-specific density) were more likely to be missed (31%-37%) or rated as equivocal (47%-50%) by HIC radiologists (77, 79). This is in line with findings from the PERFORMS program where well-defined masses and asymmetric density accounted for the highest percentage of incorrectly diagnosed cases (25%) among UK radiologists (70). This difference could be related to a large proportion of breast cancer patients in LICs in Asia that present with advanced stages compared with women in HICs. Studies in China, Taiwan, India, Vietnam (LICs and MLICs) demonstrated that the proportion of breast cancer patients with local and distant metastasis were 55% to 85% while this rate in Japan and South Korea were 40-45%, and 28%-35% in Australia, Europe, Canada and USA (59) (39, 46). Hence, radiologists in LICs and MICs with very limited breast screening abilities may not be accustomed to recalling women with small lesions or detect early cancers such as stellate lesions.

Furthermore, BREAST data has shown that Asian radiologists were more likely to achieve higher diagnostic accuracy when reading high density mammograms than mammograms with low breast density compared with their counterparts in Westernized countries. This discrepancy could be explained by the fact that Asian women tend to have smaller and higher dense breasts when viewed on mammography than Western/Caucasian women with the odds ratio for women with dense breasts versus fatty breasts increasing from 1.2 for women aged less than 45 to 1.6 for women over 65 years old according to a study of over 28,000 women of different races in the United States (80). Similarly, studies in Asian populations also support this finding with approximately 70% of mammograms in Vietnam demonstrating high breast density (81) and Chinese women had 10% higher breast density rates than Australian women (82). Thus, Asian radiologists are more likely to encounter high breast density mammographic cases compared to radiologists in Western countries where more women with low dense breasts reside.

The low level of diagnostic accuracy of the radiologists from countries with lack of breast screening programs compared with those that interpret screening cases regularly can be explained partly by the difference in expertise levels. In BREAST studies, the majority of radiologists (56%-82%) from MICs and LICs (China, Mongolia and Vietnam) reported that they read equal to or less than 20 mammograms per week whilst more than 65% of radiologists in HICs (Australia, Singapore) stated they read more than 20 cases per week. When breast screening experts interpret a mammogram, they will firstly extract information from an initial global impression, which requires a solid knowledge (also known as a memory schema) of what is normal anatomical breast features in order to differentiate abnormalities. This type of skill requires breast image readers to have considerable experience that can be achieved through conducting minimum annual readings facilitated by an active screening program (83, 84).

The number of cases read per year has been shown to be an essential component of high diagnostic performances (85). National accreditation standards in HICs such as Australia and the UK require between 2000 and 5000 reads per year (84) whereas it is lower at 960 cases every 2 years in the US. While effective training and ongoing clinical practice can develop mammographic interpretation skills, it is difficult for radiologists from LICs and MICs to achieve adequate experience in interpreting mammograms without an effective feedback loop that shows errors, successes and can offer both immediate and comprehensive feedback so that learning takes place at the point of self-testing of performance. Furthermore MIC and LIC countries tend to have shorter radiology training periods when compared to HIC such as Australia which has a 5 year radiology training period (74) (86, 87). The variation of diagnostic errors among radiologists between HICs and MICs/LICs highlights the need for effective education and training strategies tailored to better suit with local clinicians to enhance breast cancer diagnostic efficiency. One approach that could help radiologists with low levels of experience or less access to mammographic caseloads is building online interactive training platforms similar to BREAST as a continuing professional development activity. The BREAST platform currently provides the users with access to the mammogram test sets (both FFDM and DBT) at the same quality as DICOM images directly from the BREAST platform or through the PACS (Picture Archiving and Communication System). Studies have shown reasonable levels of agreement between diagnostic performances of radiologists in clinical reporting and their performance in test set environments in mammogram interpretation (69) and the use of training test sets is likely to improve diagnostic skills of radiologists in identifying abnormal lesions on screening mammograms and consequently improve patient health outcome.

The BREAST test sets, which can be made available through *via* the online platform or through workshops appended to scientific meetings/conferences has been used as an official training tool for BreastScreen Australia and BreastScreen New Zealand readers for more than a decade. Previous studies provide evidence that BREAST test sets have a positive effect on diagnostic efficacy of radiology fellows as a part of the quality assurance module of the national breast cancer screening program in Australia. Results show that the lesion sensitivity of readers recorded an increase from 20% to 31% among radiologists who read BREAST test sets regularly and this improvement was recorded in 83% of radiologists and an extraordinary 100% in radiology trainees (9). Recently, Qenam et al. (2022) reported a positive association of the improvement in positive predictive value and specificity of Australian radiologists through BREAST test sets with their diagnostic enhancement in clinical audits, further supporting the need for online educational tools like BREAST to exist (88).

Training test sets, *via* the online BREAST platform, have also been used to improve the diagnostic accuracy of radiologists from LICs. As a national example, a number of studies have been undertaken to map radiologists’ performances in reading mammograms in Vietnam (73, 89), where initial benchmarking reported that the detection of spiculated masses and stellate lesions by Vietnamese radiologists was significantly lower than calcification, discrete mass or asymmetric density (90). Therefore, Vietnamese radiologists were provided tailored BREAST training sets designed to focus on the type of lesions that they missed, with a similar level of difficulty as the pre-test set. Results showed significant improvement in diagnostic accuracy of radiologists in Vietnam, with an increase of 20.6% in the detection of stellate/spiculated mass after dedicated the training test set (90). This indicates that the cancer detection of radiologists on mammograms from less developed countries can be improved with an appropriate training intervention after areas for improvement have been mapped.

## Limitations and future opportunities

The results discussed here in relation to the BREAST program have some limitations. The majority of the test set images come from the BreastScreen Australia digital library and hence represent women that attend screening in Australia. The population with the highest participation in BreastScreen Australia is White women although Australia is a very multi-cultural country with a large migrant population from Europe and Asia, and with one in four women attending being born overseas. A small number of test sets available through the BREAST program do include images from local populations where agreements have been secured with other national institutions (such as images from Vietnam for Vietnamese test results and from Iran for Iranian results). Furthermore, a number of test sets are engineered to simulate diversified populations, such as the high-density test sets which are curated using BSA images but include women who have greater than 50% mammographic breast density. In this case, BREAST has used this collection of cases to represent an Asian population and it should be acknowledged that there are a number of limitations with this approach. The greatest authenticity comes from case collection from local populations and tested with local clinicians and education enterprises such as BREAST and others need to strive to work collaboratively with different countries and organisations to create an international radiology education community that works together yet is culturally and diversely appropriate.

Although there are obvious advantages to this online training method *via* the use of test sets, several limitations must be taken into account to consider future development. Firstly, the performances of readers were evaluated based on the gold standard set by a panel of clinical experts who curated the test sets and this is further correlated by histopathology results. However, within test sets that are designed to be completed within a reasonable timeframe for concentration, completion and feedback, there is naturally a limited number and variety of cases in the test sets which may not represent all scenarios in the screening environment. Furthermore, using the test set method might have a psychological or social desirability effect as participants are aware of being tested, and they might increase their recall rate in an attempt to maximize sensitivity. In addition, although performance within BREAST test sets do show good correlation to clinical performance, there remains the scenario that client/patient care is not affected by the choices they make within self-assessment modules or tests. Thus, the purposes of BREAST test sets are to increase diagnostic efficacy though practice, targeted learning objectives, feedback and reflection. Therefore test sets need to be incorporated into a holistic and multidisciplinary educational regime to improve expertise that also includes other documented links to improved performance, such as building a broad social learning network and participation in multidisciplinary team activities (91, 92).

Further work is needed to fully understand the precise mechanism behind the findings of why radiologists in different countries have varying performance in detecting specific types of abnormal lesions on mammograms and how clinical variability can be reduced. The power of a global breast cancer detection community that builds expertise by sharing resources from one country to another needs to continue and BREAST has achieved strong results leveraging from investment by the Australian government and assisting with mapping and improving cancer detection *via* test sets in other HIC, MIC and LIC countries. Future research and shared education might involve multi-ethnicity mammograms and eye-tracking or brain tracking technology to trace how radiologists detect lesions on mammograms so that an understanding of decision-making errors can be included. Additionally, BREAST focuses on the use of screening cases as it is intrinsically linked to the national program BreastScreen Australia, and extension of interactive learning environments is very feasible to include other imaging modalities and scenarios, such as ultrasound, MRI and contrast enhanced mammography as well as the use of artificial intelligence to predict reader error and provide personalised test sets.

In conclusion, this review showed that there was significant variation in diagnostic performances in screening mammogram test sets in different countries. Difference in breast features, discrepancies in mammogram reading experiences, the availability of high-quality national breast screening program and breast image interpretation training courses between developed and less developed countries are likely to have an impact on the variation of readers’ performances. The online educational and training methods using real-life clinical cases *via* test sets like BREAST which were shown to improve the diagnostic performances of radiologists and radiology trainees are significantly helpful to radiologists and breast image readers in different countries with and without breast screening programs in improving their diagnostic accuracy in mammogram interpretation, especially when cancer incidence rates and population demand for advanced medical imaging methods continues to rise.

## Author contributions

All authors contributed to the review conception and design. Material preparation and the first draft was performed and prepared by PT and SL. The final manuscript was written and reviewed by PT, SL, MB and CM-T. All authors read and approved the manuscript.
